# Adherence to appointments for gestational diabetes testing and experiences with two-hour postprandial glucose test: a mixed-methods study in Ghana

**DOI:** 10.1186/s12884-022-04559-5

**Published:** 2022-04-06

**Authors:** Faith Agbozo, Christina Schuler, Albrecht Jahn

**Affiliations:** 1 Department of Family and Community Health, School of Public Health, University of Health and Allied Health Sciences, Private Mail Bag, 31 Ho, Ghana; 2 Heidelberg Institute of Global Health, Heidelberg University Hospital, Heidelberg, Germany; 3 Ghana Health and Education Initiative, Sefwi Bekwai, Ghana

**Keywords:** Pregnant women, Oral glucose tolerance test, Gestational diabetes, diagnosis, Maternal health, Appointments, Booking, Schedules, Ghana

## Abstract

**Background:**

Failure to attend scheduled appointments is a common problem in healthcare. In obstetrics, diagnostic and treatment protocols for gestational diabetes mellitus (GDM) require client booking, test preparations, management and follow-up reviews. We identified the socio-demographic, obstetric and medical drivers influencing adherence to appointments for GDM testing and experiences of pregnant women’s regarding performing oral glucose tolerance test (OGTT).

**Methods:**

A convergent parallel mixed-methods study comprising a cross-sectional survey and an explorative qualitative descriptive design were used. We recruited 817 women in their first trimester of pregnancy from the antenatal clinics of primary, secondary and tertiary health facilities in Ghana. After obtaining their demographic and health history, we scheduled them for 2-h OGTT between 24 and 28 gestational weeks and estimated the odds of returning for the test. In the qualitative phase, we called 166 participants to ascertain why they failed to report. Also, we had in-depth and focused group discussions with 60 postpartum women who performed the OGTT to explore their experiences with the test.

**Results:**

Out of 817 pregnant women scheduled, 490 (59.97%) reported of which 54.59, 54.33 and 53.24% completed fasting plasma glucose, 1-h and 2-h OGTT, respectively. Maternal age above 35 years (OR: 3.56, 95% CI:1.49–8.47), secondary education (OR: 3.21, 95% CI: 1.19–8.69), formal sector employment (OR: 2.02, 95% CI: 1.16–3.51) and having same-sex children (OR: 4.37, 95% CI: 1.98–9.66) increased odds of appointment adherence whereas healthcare in a tertiary hospital (OR:0.46, 95% CI:0.22–0.96), rural residence (OR: 0.53, 95% CI: 0.34–0.85) and being overweight (OR: 0.45, 95% CI: 0.25–0.78) decreased the likelihood. Experiences were thematized into feelings about test procedure, acceptability of test, skillfulness of the health workers and information on the test. Despite the apprehension and discomforts associated with the test, the desire to know one’s disease status was the chief motivation. Empathy, reassurance and receiving ample information on the test procedures eased anxiety and improved test compliance.

**Conclusions:**

Although 40% of participants scheduled did not return, the test was generally acceptable. Socio-cultural underpinnings influenced the health-seeking behaviors, meaning that health worker interactions on test procedures need to be sensitive to the woman’s situation.

## Introduction

Non-adherence of healthcare seekers to scheduled appointments is a common problem that characterizes medical and nursing care. In the fields of oncology and obstetrics, care seekers are more health-conscious and motivated to make positive behavioral changes [1, 2]. In obstetrics, desire for quality care, positive pregnancy experience, and healthy fetal and perinatal outcomes influence pregnant women to be more compliant with the package of interventions that accompany antenatal care (ANC) [1]. However, the notion that the pregnant woman is not a patient still hinders optimum prenatal health-seeking behaviors.

Owing to its rising prevalence, many health regulators have recommended gestational diabetes mellitus (GDM) screening within 24–28 weeks of pregnancy using fasting blood glucose, or two-hour oral glucose tolerance test (OGTT) considered as the gold standard [3–7]. Adding GDM testing to the cascade of ANC package, although challenging, especially for low- and middle-income countries (LMICs), is necessary to reduce the risk of adverse pregnancy outcomes [8, 9] and minimize associated long-term cardio-metabolic complications [10, 11].

In many LMICs, besides the challenge of few and late ANC contacts, is non-adherence to essential medical services. Non-adherence here refers to the situation whereby clients are scheduled for a follow-up consultation or diagnostic procedure for which they agreed to attend, yet fail to show up. For example, in northern Tanzania, 23% of pregnant women who were screened and booked to return between the next day and 1 month for fasting plasma glucose (FPG) and OGTT did not return [12]. Assuming the 19.5% GDM prevalence observed among those wo tested was the same as for the 100 who did not return, 23 additional pregnant women would be diagnosed, implying a missed opportunity to receive treatment [12]. In South Africa, 35.7% (*n* = 1000) of pregnant women scheduled for GDM testing did not perform the test due to unreachable telephone lines (*n* = 194) and relocation from the area (*n* = 163) [13]. However, demand-side factors, including cost obligations, obstetric history, transportation constraints, support systems and socio-cultural practices cold hinder the process [14, 15].

Studies have investigated women’s experiences with GDM screening [16, 17] and the psychological effect of a positive diagnosis on pregnancy outcome [1, 18]. However, socio-demographic background of pregnant women who honor appointments vis-à-vis the defaulters is unknown, so are the factors that influence adherence to appointments for GDM testing and experiences with OGTT. As the prevalence of GDM keeps rising globally, knowledge derived through this mixed methods approach will aid in planning interventions to improve compliance with appointments not only for GDM testing, glycemic management and post-delivery follow-up, but also strengthen general medical and nursing care. Our study sought to (1) characterize the pregnant women who did not show up for the scheduled GDM diagnostic testing and compare with those who reported (2); assess the determinants and actual reasons for not honoring the appointment and (3); explore experiences related with performing 2-h postprandial blood glucose test during pregnancy.

## Methodology

### Mixed methods design

We used the convergent parallel mixed-methods design focusing on the follow-up sequential explanatory model [19]. This study was part of an observational study intended to validate the diagnostic accuracy of instruments for screening and diagnosing GDM. The qualitative phase was conducted post-delivery but was integrated into the quantitative research. The data was analyzed independently and interpreted jointly.

### Study setting

Pregnant women were recruited from the antenatal clinic of one teaching hospital, three municipal hospitals and one health centre in the Volta Region, Ghana. These facilities provide care at the tertiary, secondary and primary levels, respectively. The tertiary health facility provided all the range of emergency obstetric care services, the secondary facilities provided all the basic and some selected comprehensive emergency obstetric care services, whilst the primary health facility provided only the basic services. Cumulatively, the five study facilities have a bed capacity of 745 and provide antenatal, delivery and postpartum services. In the region, there are 1,098,854 females of reproductive age (15–49 years) who reside in rural (66.3%) and urban areas (37.7%). The policy on GDM diagnosis in Ghana follows the ‘one-step’ universal screening approach targeted at all pregnant women, regardless of their disease risk status [20].

### Quantitative methods

#### Design

The quantitative design was a cross-sectional survey incorporated into a prospective study whereby pregnant women were scheduled to perform all the screening (urine glycosuria, random blood sugar, risk stratification) and diagnostic tests for GDM (fasting plasma glucose, one and two-hour OGTT).

#### Sample

A sample size of 768 pregnant women was estimated. Basically, we applied a target population of ≈500,000 women in their reproductive age (15–49 years) in the study area; a 95% confidence level corresponding to 1.96 alpha, 0.05 error margin, a default population proportion of 50% and doubling of the resultant sample size to account for drop-outs. Women who met the eligibility criteria (no history of diabetes, gestational age below 13 weeks and maternal age above 15 years but no upper age limit) were consecutively recruited.

#### Data collection

The procedure for data collection have been detailed elsewhere [21]. Essentially, 817 pregnant women in the first trimester were selected to follow the universal screening process for GDM irrespective of their risk stratum. Recruitment in the first trimester helped methodologically to decipher and exclude participants having pre-existing diabetes from gestational diabetes and afforded ample time to educate and prepare the pregnant women for the GDM testing. Up until 20 gestational weeks, we conducted face-to-face interviews to obtain details on participants socio-demographic background, obstetric and medical history. Between 24 to 28 gestational weeks, we scheduled all the participants for FPG, 1-h and 2-h OGTT. All participants were called the night before the test and reminded of the appointment and the test preparation (overnight fasting and timely arrival at the laboratory). GDM diagnosis was based on the IADPSG/WHO diagnostic criteria [5, 7].

#### Analysis

Quantitative analysis was conducted in Stata software (version 14.2). The study participants were stratified into two groups according to whether they showed up for the GDM testing or not. Between-group differences were tested using Chi-square test for categorical variables and Student’s t-test for continuous variables. We performed a univariate binary logistic regression analysis to estimate the odds ratio associated with showing up for the appointment. The outcome measure was binary in nature – adherence and non-adherence to the OGTT appointment schedule. We performed a univariate logistic regression to identify the socio-demographic, medical and obstetric factors associated with GDM. Variables included in the univariate (unadjusted) analysis are presented in Tables 3 and 4. In terms of the multivariate model, variables that were either known from literature to be linked with adherence to hospital appointments or had a *p*-value < 0.300 were selected. Some socio-demographic variables that we considered for inclusion in the multivariate analysis included possession of a mobile phone, place of residence, ethnicity, level of education and type of occupation. The obstetric/medical variables comprised sex of children born, the experience of birth complications, previous surgery, positive urine glucose and urine protein and medical history of diabetes, hypertension and obesity. We adjusted for maternal age, parity, level of care and gestational age at first ANC booking. Adjusted odds ratio (OR) associated with attending the diagnostic appointment were reported with the corresponding 95% confidence interval.

### Qualitative design, participants, interviews and analysis

#### Design

The qualitative explorative descriptive design, particularly relevant in healthcare research to explore how patients experience illness and medical interventions, was employed [22–25]. The focus was to generate data that describe the ‘what of experiences’ from a subjective perspective [23]. The aim was to understand the experiences of individual women who underwent the OGTT in their unique context.

#### Participants

During the postpartum period, we tried calling all the 435 participants who completed the 2-h OGTT. Luckily, 102 agreed to provide information regarding their experiences with the GDM tests they performed. Overall, 48 in-depth interviews and one focus group discussion (FGD) involving 12 participants were successively conducted by which time data saturation was reached, requiring termination of the remaining interviews planned.

#### Data collection

On the day of the test, we had brief telephone interviews with a section of the pregnant women who failed to show up for the tests to understand why they did not honor the invitation. During the test procedures, we took field notes in line with observations made. Aided by a semi-structured topic guide, two health personnel conducted the interviews during the postnatal period. The one-on-one interviews were conducted during routine postnatal visits. The venue was the same health facility where the women had received antenatal care for the index pregnancy. But in a few cases, the interviews were done in the womens’ homes, based on their preference. The FGD on the other hand, was conducted in a health facility that was within reach for majority of the women. The one-on-one interviews were conducted in two local languages and sparingly in English, whereas the FGD was conducted in Ewe. The topic guide covered the women’s experiences regarding the GDM test they did. Probing questions mainly focused on the glucose solution, sample taking procedures and support services provided by the health personnel. Participants recruitment continued until extra interviews yielded no new information [26, 27].

#### Data analysis

The interviews were audio-recorded and transcribed verbatim. Field notes from observations and short informal discussions with participants during the test procedures were used to triangulate the data. Based on Erlingsson and Brysiewicz’s [28] procedure, a content analysis was done using Atlas.ti (Version 8.4). Qualitative descriptive research is often data-driven, and because few studies have investigated this phenomenon, an inductive approach was used to combine fragmented observations into a more general statement to ensure that findings are understandable and applicable to healthcare practice [22, 29, 30]. Initially, the data was read several times. Thereafter, it was divided into units of meaning that were condensed. The condensed units were then abstracted and labelled with codes. One author independently assessed the explanatory value of the developing codes and categories against the transcripts. Diverging codes were re-evaluated until consensus was reached. The codes were later grouped into categories and sub-categories. After discussing the categories, four main, and eleven sub-categories emerged. All the authors read, discussed and agreed on the final categorization.

#### Rigor

Lincoln and Guba’s [31] criteria of trustworthiness was followed to safeguard quality control and ensure that the results could be transferable to similar contexts. Issues relating to dependability and confirmability were addressed using audit trail that incorporated voice records, transcripts, field observations and notes. Confirmability was further achieved through a systematic treatment of the data, with repeated readings to help grasp the content, and careful generation of the categories to reflect the participants’ voices. The results were vividly described, and verbatim quotations were used to clarify the interviewees’ experiences. This way, transferability was ensured.

## Results

### Quantitative results

Out of the 817 pregnant women we scheduled for GDM testing between 24 to 28 gestational weeks, 490 (59.98%) reported to the health facility to conduct the tests. However, 44 of the 490 pregnant women representing 8.98%, came in a non-fasting state and were thus excluded. Overall, the pregnant women performed pre-prandial (fasting plasma) glucose test (*n* = 446), 1-h (*n* = 445) and 2-h OGTT (*n* = 435) postprandial glucose tests. When we called the 327 participants who failed to report for the test, the lines of 161 were unreachable. The 166 participants we were able to reach gave diverse reasons for not showing up. These included relocation miles away from the study area (*n *= 60), losing interest in continuing as a study participant (*n *= 46) and finding the appointment date no longer convenient (*n *= 34). Twenty either forgot about the appointment or had no tangible reason, whereas six lost the pregnancy.

Comparing the two groups of participants who returned and did not return for the appointment, we observed significant differences in maternal age, level at which healthcare was provided, place of residence, ethnicity and spouse’ occupation (Table 1). Participants who did not honor the appointment were significantly younger, had more induced pregnancy terminations and conducted the GDM screening test much later in pregnancy (Table 2). For polychotomous variables with multiple response levels, we used Bonferroni adjustments to identify the groups contributing significantly to the associations (Table 1). We found that participants within 20–24 and ≥ 35 years age brackets, primary healthcare facility users (16.5% vs 13.8%), women who were indigenes of the study area and those whose partners had secondary education (34.8% vs 27.4%) or were formal sector employees (24.0% vs 32.0%) contributed to the effect size.

**Table 1:** Cross-tabulation showing socio-demographic characteristics of the pregnant women categorized according to adherence to the appointment for GDM testing

Variable	Sub-scale	Not reported n (%) (***n*** = 337)	Reported n (%) (***n*** = 490)	Overall N (%) (***N*** = 827)	***P***-value
Maternal age (years)	≤19	35 (10.9)	35 (7.2)	70 (8.7)	**0.024**
	20–24 ^a^	91 (28.3)	105 (21.6)	196 (24.2)	
	25–29	89 (27.6)	146 (30.0)	235 (29.0)	
	30–34	66 (20.5)	112 (23.0)	178 (22.0)	
	≥35 ^a^	41 (12.7)	89 (18.3)	130 (16.1)	
Level of care	Primary ^a^	33 (9.8)	81 (16.5)	114 (13.8)	**0.022**
	Secondary	257 (76.3)	346 (70.6)	603 (72.9)	
	Tertiary	47 (13.9)	63 (12.9)	110 (13.3)	
Mobile phone	Personal phone	285 (84.6)	437 (89.2)	722 (87.3)	0.146
	Household	21 (6.2)	22 (4.5)	43 (5.2)	
	None	31 (9.2)	31 (6.3)	62 (7.5)	
Place of residence	Urban ^a^	179 (54.1)	311 (66.2)	490 (61.2)	**< 0.0001**
	Rural ^a^	152 (45.9)	159 (33.8)	311 (38.8)	
Religion	Christian	307 (91.1)	428 (92.4)	735 (91.9)	0.514
	Moslem	30 (8.9)	35 (7.6)	65 (8.1)	
Ethnicity	Ewe ^a^	246 (73.4)	311 (66.5)	557 (69.4)	**0.007**
	Guan ^a^	29 (8.7)	76 (16.2)	105 (13.1)	
	Other tribes ^b^	60 (17.9)	81 (17.3)	141 (17.6)	
Marital status	Married	235 (71.0)	333 (73.2)	568 (72.3)	0.239
	Cohabitating	45 (13.6)	70 (15.4)	115 (14.6)	
	Single	51 (15.4)	52 (11.4)	103 (13.1)	
Woman’s education	Primary	59 (17.8)	69 (14.9)	128 (16.1)	0.727
	Junior high ^c^	157 (47.4)	232 (50.1)	389 (49.0)	
	Senior high ^c^	68 (20.5)	96 (20.7)	164 (20.7)	
	Tertiary	47 (14.2)	66 (14.3)	113 (14.2)	
Partner’s education	Primary	27 (8.2)	41 (9.0)	68 (8.7)	0.142
	Junior high ^c^	115 (35.1)	168 (36.8)	283 (36.1)	
	Senior high ^a c^	114 (34.8)	125 (27.4)	239 (30.5)	
	Tertiary	72 (22.0)	122 (26.8)	194 (24.7)	
Woman’s occupation	Unemployed	78 (23.1)	85 (18.3)	163 (20.3)	0.211
	Informal sector	218 (64.7)	314 (67.5)	532 (66.3)	
	Salaried worker	41 (12.2)	66 (14.2)	107 (13.3)	
Partner’s occupation	Unemployed	24 (7.3)	20 (4.3)	44 (5.6)	**0.019**
	Informal sector	226 (68.7)	293 (63.7)	519 (65.8)	
	Salaried worker ^a^	79 (24.0)	147 (32.0)	226 (28.6)	
Household size	1–5 members	245 (81.9)	362 (84.4)	607 (83.4)	0.419
	≥6 members	54 (18.1)	67 (15.6)	121 (16.6)	

^a^Bonferroni adjustment showing the column proportions that differ significantly

^b^Other tribes include Akan, Ga, northern tribes and foreigners

^c^Junior and senior high are equivalent to the 8th and 12th grades, respectively

**Table 2:** T-test showing mean socio-demographic, obstetric and medical characteristics of the pregnant women categorized according to adherence to the appointment for GDM testing

Variables	Not reported Mean (SD)	Reported Mean (SD)	Overall Mean (SD)	***P***-value
Maternal age (years)	26.95 (6.20)	28.33 (6.20)	27.78 (6.239)	0.002
Gestational age (weeks) ^a^	17.15 (7.08)	14.52 (6.64)	15.64 (6.95)	< 0.0001
Persons in household	3.98 (2.28)	3.96 (2.27)	3.97 (2.27)	0.888
Parity (live children)	1.33 (1.35)	1.45 (1.28)	1.40 (1.31)	0.226
Gravida (pregnancies)	2.71 (1.66)	2.73 (1.49)	2.72 (1.56)	0.854
Number of miscariages ^b^	0.90 (1.13)	0.60 (0.80)	0.71 (0.95)	0.002
Random sugar (mmol/l) ^c^	5.02 (1.10)	5.17 (1.10)	5.11 (1.10)	0.064
Systolic BP (mmHg) ^c^	107.88 (11.64)	108.05 (11.48)	107.98 (11.54)	0.844
Diastolic BP (mmHg) ^c^	65.92 (9.71)	65.38 (9.36)	65.61 (9.50)	0.431
Weight (kg) ^c^	62.01 (12.59)	62.38 (12.94)	62.22 (12.79)	0.675
Height (cm) ^c^	160.83 (9.38)	162.73 (7.88)	161.94 (8.58)	0.003
Body mass index (kg/m^2^)	24.01 (5.11)	23.47 (4.96)	23.69 (5.03)	0.154

^a^This is the gestational age when screening for GDM was done

^b^This includes both spontaneous and induced abortions

^c^These measurements were taken in the first trimester

*BP* blood pressure, *kg* kilogram, *cm* centimetre

In the univariate analysis (Table 3), we observed that advanced maternal age and receiving care in lower-level facilities increased the odds of showing up for the appointment. Contrarily, rural dwelling, being an indigene, unemployed or informal sector worker reduced the odds of showing up. In terms of the obstetric and medical factors, we observed in the univariate regression (Table 4) that participants who were screened for GDM in the first trimester or experienced previous delivery complications had higher odds of showing up. In contrast, primiparous and overweight women were less likely to show up.

**Table 3:** Univariate binary logistic regression showing the socio-demographic factors associated with adhering to the appointment for GDM diagnosis

Variable	Reference	Sub-scales	uOR	95% CI	SE	***P***-value
Maternal age (years)	< 20 years	20–24	1.15	0.66–1.99	0.32	0.608
		25–29	1.64	0.95–2.80	0.44	0.071
		30–34	1.69	0.97–2.96	0.48	0.064
		≥35	2.17	1.19–3.94	0.66	0.011
Level of care	Secondary	Primary care	1.82	1.17–2.81	0.40	0.007
		Tertiary care	0.99	0.66–1.50	0.20	0.983
Mobile phone	Own phone	In household	0.68	0.36–1.26	0.21	0.226
		None	0.65	0.38–1.09	0.17	0.107
Residence	Urban	Rural	0.60	0.45–0.80	0.08	0.001
Ethnicity	Non-indigenes	Indigenes	0.71	0.52–0.97	0.11	0.035
Religion	Christian	Moslem	0.83	0.50–1.39	0.21	0.493
Marital status	Co-habiting	Married	0.91	0.60–1.37	0.19	0.656
		Single	0.65	0.38–1.12	0.17	0.124
Woman’s education	Primary	Junior high	1.26	0.84–1.88	0.25	0.254
		Senior high	1.20	0.75–1.92	0.28	0.429
		Tertiary	1.20	0.72–2.01	0.31	0.483
Partner’s education	Primary	Junior high	0.96	0.56–1.65	0.26	0.888
		Senior high	0.72	0.41–1.24	0.20	0.244
		Tertiary	1.11	0.63–1.96	0.32	0.704
Woman’s occupation	Salaried worker	Unemployed	0.67	0.41–1.11	0.17	0.123
		Informal job	0.89	0.58–1.37	0.19	0.609
Partner’s occupation	Salaried worker	Unemployed	0.44	0.23–0.86	0.14	0.016
		Informal job	0.69	0.50–0.96	0.11	0.029
Household size	1–5 members	≥6 members	0.83	0.56–1.24	0.16	0.384

*uOR* Unadjusted odds ratio, *CI* Confidence interval, *SE* Standard error

**Table 4:** Univariate logistic regression showing the obstetric and medical factors associated with returning for the GDM diagnostic testing

Variable	Sub-scales	Overall N (%)	uOR	95% CI	SE	***P***-value
Gestation age at screening ^a^	< 12 weeks	275 (39.7)	1.85	1.35–2.54	0.29	*< 0.0001*
Gravida ^b^	Primigravida	193 (24.5)	0.78	0.56–1.09	0.13	0.158
Parity	Nullipara	219 (29.3)	0.64	0.41–0.99	0.14	*0.045*
	1–2	385 (51.5)	0.82	0.55–1.23	0.16	0.357
	3+	143 (19.1)	Ref			
Sex of children	Males only	159 (19.9)	1.07	0.70–1.63	0.23	0.750
	Females only	155 (19.4)	1.38	0.89–2.14	0.30	0.139
	Both sexes	198 (24.8)	Ref			
Pregnancy planned	Yes	486 (60.8)	1.03	0.77–1.37	0.15	0.820
Glad about pregnancy	Yes	729 (91.5)	1.26	0.76–2.08	0.32	0.357
Body mass index	Underweight	85 (11.50)	0.73	0.46–1.18	0.17	0.208
	Normal	404 (54.67)	Ref			
	Overweight	167 (22.60)	0.63	0.44–0.91	0.11	*0.014*
	Obese	83 (11.23)	0.77	0.48–1.25	0.19	0.307
Previous delivery place ^c^	Hospital	397 (49.5)	1.12	0.74–1.70	0.23	0.572
History of marriages	Yes	117 (14.1)	1.04	0.64–1.68	0.25	0.856
Pregnancy complications ^d^	Yes	11 (1.4)	1.05	0.30–3.68	0.67	0.937
Delivery complications ^f^	Yes	30 (3.7)	3.16	1.18–8.45	1.58	*0.021*
Prior cesarean section	Yes	62 (8.0)	1.33	0.75–2.37	0.39	0.319
Any child dead	Yes	43 (5.4)	1.13	0.60–2.14	0.36	0.691
Dipstick urine glucose	Trace +	35 (4.3)	0.55	0.28–1.09	0.19	0.091
Dipstick urine protein	Trace +	165 (22.1)	1.23	0.86–1.76	0.22	0.250
Sickle cell disease	Positive	24 (3.0)	1.44	0.61–3.41	0.63	0.403
Asthma	Yes	16 (2.0)	1.58	0.54–4.61	0.86	0.395
Diabetes	Yes	4 (0.5)	2.15	0.22–20.85	2.49	0.506
Diabetes (family)	Yes	47 (5.9)	1.15	0.63–2.12	0.35	0.634
Hypertension	Yes	29 (3.6)	1.17	0.54–2.53	0.45	0.672
Hypertension (family)	Yes	143 (17.8)	0.85	0.59–1.23	0.15	0.406
Previous surgery	Yes	32 (4.0)	2.18	0.96–4.92	0.90	0.060

*uOR* Unadjusted odds ratio, *CI* Confidence interval, *SE* Standard error

The reference variables were ^a^screening for GDM after 13–20 gestational weeks; ^b^multigravidae; and ^c^previous delivery at home or with a traditional birth attendant. The ^d^pregnancy complications included miscarriages, antepartum haemorrhage and pregnancy-induced hypertension, while the ^f^delivery complications included stillbirth, postpartum haemorrhage, placental previa, prolonged labour and retained placenta

Adjusting for covariates, maternal age between 25 and 29 years (OR: 2.10, 95% CI: 1.16–3.79) and ≥ 35 years (OR: 3.56, 95% CI: 1.49–8.47), secondary education (OR: 3.21, 95% CI: 1.19–8.69), partner’s employment in the formal sector (OR: 2.02, 95% CI: 1.16–3.51), GDM screening in the first trimester (OR: 1.73, 95% CI: 1.10–2.72) and having only male (OR: 2.64, 95% CI: 1.25–5.58) or only female children (OR: 4.37, 95% CI: 1.98–9.66) significantly increased the odds of attending the appointment. Conversely, tertiary hospital users (OR: 0.46, 95% CI: 0.22–0.96), rural dwellers (OR: 0.53, 95% CI: 0.34–0.85) and overweight women (OR: 0.45, 95% CI: 0.25–0.78) were less likely to show-up (Table 5).

**Table 5:** Multivariate logistic regression showing the factors associated with returning for GDM test

Variable	Reference	Sub-scales	aOR	95% CI	SE	***P***-value
Maternal age	20–24	< 19	1.73	0.71–4.19	0.78	0.224
		25–29	2.10	1.16–3.79	0.63	0.014
		30–34	1.96	0.99–3.89	0.68	0.054
		≥35	3.56	1.49–8.47	1.57	0.004
Level of care	Secondary	Tertiary	0.46	0.22–0.96	0.17	0.033
		Primary	1.33	0.66–3.16	0.51	0.452
Mobile phone	Own phone	In household	0.64	0.23–1.76	0.33	0.390
		No phone	0.65	0.18–2.25	0.41	0.502
Place of residence	Urban	Rural	0.53	0.34–0.85	0.12	0.007
Ethnicity	Indigenes	Non-indigenes	1.41	0.84–2.36	0.37	0.189
Education	Tertiary	Primary	2.40	0.78–7.40	1.37	0.125
		Junior high	3.21	1.19–8.69	1.63	0.021
		Senior high	2.19	0.84–5.71	1.07	0.109
Occupation	Informal	Unemployed	0.83	0.45–1.53	0.25	0.561
		Salaried job	1.46	0.56–3.77	0.71	0.430
Partner’s occupation	Informal	Unemployed	1.13	0.42–3.01	0.56	0.805
		Salaried job	2.02	1.16–3.51	0.57	0.013
Gestational age	13–20 weeks	< 13 weeks	1.73	1.10–2.72	0.39	0.017
Parity	Nullipara	1–2	1.16	0.36–3.68	0.68	0.796
		3–4	2.34	0.60–9.18	1.63	0.220
		5+	0.42	0.061–2.90	0.415	0.382
Children’s sex	Both sexes	Males only	2.64	1.25–5.58	1.00	0.011
		Females only	4.37	1.98–9.66	1.76	< 0.0001
Birth complications	No	Yes	2.75	0.64–11.68	2.03	0.169
Previous surgery	No	Yes	3.51	0.88–13.91	2.46	0.073
Urine glucose	Negative	Trace +	0.54	0.20–1.47	0.27	0.232
Urine protein	Negative	Trace +	1.59	0.95–2.66	0.41	0.077
Diabetes	No	Yes	0.47	0.037–5.99	0.61	0.564
Hypertension	No	Yes	2.07	0.56–7.71	1.39	0.275
Body mass index	Normal	Underweight	0.67	0.35–1.31	0.23	0.247
		Overweight	0.45	0.25–0.78	0.12	0.005
		Obese	0.53	0.25–1.12	0.20	0.099

*aOR* Adjusted odds ratio, *CI* Confidence interval, *SE* Standard error

Model summary: number of observations = 480; LR chi^2^(36) = 84.93; Prob > chi^2^ = 0.0000, Pseudo R^2^ = 0.1349; Log likelihood = − 272.41793

### Qualitative results

Analysis of the qualitative data revealed four major themes that described participants’ experiences about the test protocol and their views about the test itself. The themes are feelings about the test, acceptability of preliminary preparations and test procedures, professionalism of health workers and adequacy of information received regarding the test.

### Feelings about the test

The majority reacted positively towards the test procedure, as they found it uncomplicated and were willing to repeat the test in subsequent pregnancies. A few were scared because they did not fully understand why their blood was drawn, while some lamented that the amount drawn was too much and could affect their fetus’ growth and development as well as their own health. Nonetheless, performing the test was deemed relieving as it helped to know their disease status, implying abnormalities could be detected early and treated. They bemoaned that the test was not done routinely and requested that every pregnant woman be given the opportunity.

*“As for the test, it is good. If you will continue to do it … … . it should be done for all pregnant women who go for antenatal.”* [Participant, IW 15]

### Acceptability of test procedures

Participants had difficulty fasting overnight. In fact, some either forgot or deliberately decided not to fast. Complaints of dizziness, tiredness and weakness were attributed to the overnight fast. We observed symptoms of fainting such as frequent yawning, lightheadedness, weakness and profuse sweating. The ingestion of 75-g glucose dissolved in 300 ml of water at room temperature was thought to be sugary tasting, voluminous and cold. It triggered nauseous sensations, whereas some vomited during or after drinking the glucose solution. As a result, some pregnant women took approximately 10 min to drink the solution. The waiting time required to complete the test (averagely 2.5 h) was met with some visible irritability, which was sequel to the opportunity cost of having to miss work. However, those who appreciated the test regimen had little reservations.

*“ … .because it was three tests that it was done so it is normal if we spent that time. If it was just one test that took that much time it would have been bad but since it is three of them … .. it's okay.”* [Participant, IW 19]

### Professionalism of health workers

Pain associated with venipuncture was expected. The majority understood its necessity and psyched themselves up for it by taking their attention off the phlebotomy. Occasionally, the phlebotomists struggled to locate the veins but soothed the women through quality care, reassurance and empathy. Compared with routine care, participants expressed satisfaction, especially with the respectful care received.

*“I think you treated me well, better than when I went to [mentioning the hospital] because when I came to you people, I was given time to rest and returned after an hour. Even when people started feeling dizzy, you people you were encouraging them”* [Participant, IW 46]

### Information on the test

A few mothers complained of not receiving detailed education about the test thereby not amply understanding its essence. Participants were informed that only those whose blood glucose values were abnormal would be notified regarding feedback on the test results. However, many were displeased with that approach. The women reiterated their desire for better information on the test procedure and outcome, alongside nutrition in pregnancy, GDM prevention measures, and the management regimen.

*“We were expecting the result so that if there is any problem, we will know the kind of things we should eat, … how to go about the changes because when you are pregnant, there are some things you can't eat and there are some things you should just eat in small quantities.”*[Participant, IW 29]

## Discussion

In the wake of the increasing prevalence of GDM globally, prompt screening is crucial for its management and reduction of complications. However, the failure of pregnant women to adhere to protocols for GDM detection and management is not uncommon [12, 13, 32, 33]. Yet, conducting OGTT is described as worthwhile because treatment can be initiated in the worst case of hyperglycemia [16]. Similar to our participants’ request, women in both developed [16] and developing countries  [32] have expressed the desire for OGTT be extended to be all pregnant women. Their quest for an expanded screening means that in implementing the universal screening approach for GDM detection, pregnant women are more likely to accept the test. However, many LMICs are still screening selectively [33], making it seem as though GDM assessment is a privilege.

Complaints of nausea and vomiting after drinking the glucose solution, dizziness and hunger resulting from the overnight fast, phlebotomy pain, long test period (2–3 h), overcrowding at the laboratory, and uncomfortable waiting area are not new [16, 32, 34–36]. In Morocco, nine out of 455 pregnant women who ingested the 75-g dextrose vomited [37]. In Germany, pregnant women were willing to recommend OGTT to others despite complaints of dizziness, unpleasant taste of the glucose solution, long waiting time and perceived increased fetal movement after taking the glucose [16]. Innovations such as adding lemon drops to the glucose solution have been reported to alleviate the nausea [17].

Concerns about the test adversely affecting the mother and fetus and the perceived high quantum of blood drawn are not limited to our study [17, 36]. In India, pregnant women have complained about voluminous blood taken when anemia is highly prevalent [17]. Effective patient-health provider interaction could allay such anxiety. The quest for comprehensive education on OGTT is widespread, rightly justified and cannot be ignored, as traditionally, counseling on GDM is inadequate [14]. In this regard, when patient education is strengthened and their emotional needs addressed, compliance with the diagnostic and treatment protocols could be enhanced [33, 38], thereby minimizing the associated depressive, anxiety and stress symptoms [39]. Involving significant others in the care process, providing educational materials and arousing enthusiasm amongst pregnant women, their families, health professionals and policymakers on the seriousness of GDM is advised [34, 40]. Also, modalities for follow-up GDM test should be discussed and integrated into the ANC plan.

OGTT is expensive, requires test preparation, not physiologic, ethnicity-dependent, unpleasant and given without consideration to body weight [12, 37, 41, 42]. Staff shortage, overcrowding and long waiting periods characterize healthcare in LMICs. The overnight fasting is challenging [33, 36]. Meanwhile, prolonged fasting can alter fluid state [43], catabolism and insulin response [44]. In parts of India, pregnant women perform 2-h OGTT irrespective of their fasting state [45]. As health services in most LMICs are generally walk-in, many care-seekers are not familiar with appointments. Eliminating the overnight fasting component will widen the GDM test net. However, this should be informed by the health system context.

Our quantitative findings reaffirmed some established socio-demographic, obstetric and health system factors which facilitate adherence. Rural dwelling has been linked with infrequent [46] and late utilization of ANC services [14, 15, 34]. Rural inhabitants are often disadvantaged socioeconomic-, information- and infrastructure-wise. They invest more money, time and effort to access GDM services which are usually rendered in tertiary and specialist facilities [33]. As ANC is delivered mainly at the primary care level, participants attending tertiary hospitals are referred patients who often reside far from the referral facility, explaining why rural dwellers had a higher non-adherence rate. Also, willingness to initiate and complete GDM test varies according to access to infrastructure, counseling on the test, test characteristics, timing and clinic hours [17]. Women above 34 years were more responsive because they have more autonomy. In Tanzania, women who flouted the appointment were unmarried [12]. Lack of access to telephone lines and relocation from the study area were a hindrance in South Africa [13]. Although we did not find mobile phone ownership as a significant determinant, unreliable mobile network limited the number of participants reached.

Interestingly, having same-sex children and being overweight were negative correlates. Multiparous women who had same-sex children were more likely to meet the schedule, but the probability was higher among women who had only female (OR = 4.37) compared to only male (OR = 2.64) children. Findings in this context are limited, but couples’ desire to have gender-balance or male children is documented [47, 48]. Overweight women were also less likely to report. Body image perceptions are deeply rooted in socio-cultural norms and influences health-seeking behaviors [35]. In many low-income settings, the overweight body size is socially preferred and indicates wealth, marital bliss, beauty and health [49]. Although this perception is changing, it is slower in rural areas. This buttresses the need to heighten education on the health risk associated with obesity. Educational interventions should be socio-culturally sensitive, woman- and family-centered and embedded in the continuum of the care plan.

### Strengths and limitations

Using the mixed-methods approach, subtle issues that are neither verbalized nor consciously known to influence adherence to GDM diagnosis emerged. Calling the non-attendees helped to understand why they did not report. Generally, women are likely to comply with any medical intervention they believe will contribute to the health and well-being of mother-offspring, irrespective of the associated discomforts. While this is a strength to leverage, it can affect the interview responses. The qualitative findings should be interpreted bearing this in mind. OGTT is not a routine ANC practice in many LMICs. It was conducted in a study rather than a clinical context. While some participants might feel fortunate to have done the test at no cost, others could interpret it as an optional test done at the women’s discretion. The second scenario might reduce test compliance. Therefore, it is likely that compliance will be better in a clinical setting. Finally, women who did the GDM test differed significantly in terms of age, height, gestational age of pregnancy at GDM screening, place of residence, ethnicity and partner’s occupation. These issues should need consideration when planning interventions.

### Conclusions and implications for practice

As social norms drive health-seeking behaviors, clinically meaningful, socio-culturally relevant, and woman-centered uniqueness should underpin health worker-patient interactions. Bridging the access inequity gap should be paramount during patient teaching concerning the diagnostics and management regimen for hyperglycemia in pregnancy and maternal health care in general. Strategies to strengthen adherence to schedules for gestational diabetes will require integrating the diagnosis and treatment support into primary healthcare. This will bring health services closer to rural inhabitants while also reducing referral to higher healthcare levels where non-attendance is most likely. This will be a step towards attaining universal health coverage. It is vital to publicize that chronic disease status entails long-term care and follow-up appointments if a positive treatment outcome is to be achieved.

## Data Availability

The datasets analyzed during the current study is part of a longitudinal study. The dataset is available from the corresponding author on reasonable request.
